# Invasive Brain–Computer Interface for Communication: A Scoping Review

**DOI:** 10.3390/brainsci15040336

**Published:** 2025-03-24

**Authors:** Shujhat Khan, Leonie Kallis, Harry Mee, Salim El Hadwe, Damiano Barone, Peter Hutchinson, Angelos Kolias

**Affiliations:** 1 Department of Clinical Neuroscience, University of Cambridge, Cambridge CB2 1TN, UK; sk2361@cam.ac.uk (S.K.); hwjm2@medschl.cam.ac.uk (H.M.); se471@cam.ac.uk (S.E.H.); dgb36@cam.ac.uk (D.B.); pjah2@cam.ac.uk (P.H.); 2 Department of Medicine, University of Cambridge, Trinity Ln, Cambridge CB2 1TN, UK; leonie.kallis1@nhs.net; 3 Department of Rehabilitation, Addenbrookes Hospital, Hills Rd., Cambridge CB2 0QQ, UK; 4 Bioelectronics Laboratory, Department of Electrical Engineering, University of Cambridge, Cambridge CB2 1PZ, UK; 5 Department of Neurosurgery, Houston Methodist, Houston, TX 77079, USA; 6 Department of Neurosurgery, Addenbrookes Hospital, Hills Rd., Cambridge CB2 0QQ, UK

**Keywords:** brain–computer interface, brain–machine interface, BCI, neurotechnology, paralysis, ALS, stroke, spinal cord, ECoG, multielectrode array, stereoelectroencephalography

## Abstract

Background: The rapid expansion of the brain–computer interface for patients with neurological deficits has garnered significant interest, and for patients, it provides an additional route where conventional rehabilitation has its limits. This has particularly been the case for patients who lose the ability to communicate. Circumventing neural injuries by recording from the intact cortex and subcortex has the potential to allow patients to communicate and restore self-expression. Discoveries over the last 10–15 years have been possible through advancements in technology, neuroscience, and computing. By examining studies involving intracranial brain–computer interfaces that aim to restore communication, we aimed to explore the advances made and explore where the technology is heading. Methods: For this scoping review, we systematically searched PubMed and OVID Embase. After processing the articles, the search yielded 41 articles that we included in this review. Results: The articles predominantly assessed patients who had either suffered from amyotrophic lateral sclerosis, cervical cord injury, or brainstem stroke, resulting in tetraplegia and, in some cases, difficulty speaking. Of the intracranial implants, ten had ALS, six had brainstem stroke, and thirteen had a spinal cord injury. Stereoelectroencephalography was also used, but the results, whilst promising, are still in their infancy. Studies involving patients who were moving cursors on a screen could improve the speed of movement by optimising the interface and utilising better decoding methods. In recent years, intracortical devices have been successfully used for accurate speech-to-text and speech-to-audio decoding in patients who are unable to speak. Conclusions: Here, we summarise the progress made by BCIs used for communication. Speech decoding directly from the cortex can provide a novel therapeutic method to restore full, embodied communication to patients suffering from tetraplegia who otherwise cannot communicate.

## 1. Introduction

Communication is a vital part of social interaction that can be impaired following conditions such as stroke, trauma, and neuromuscular diseases (NMDs). For many patients, the deprivation of communication is severely debilitating and significantly worsens the quality of life [1]. In the most severe of cases, patients can present with a profound state of paralysis, characterised by tetraplegia, cranial nerve dysfunction, and anarthria, yet with preserved cognitive function, known as the locked-in syndrome (LIS) [2]. As such, the development and implementation of functional communication systems for this population is a clinical and research priority [3].

**Figure 1:**
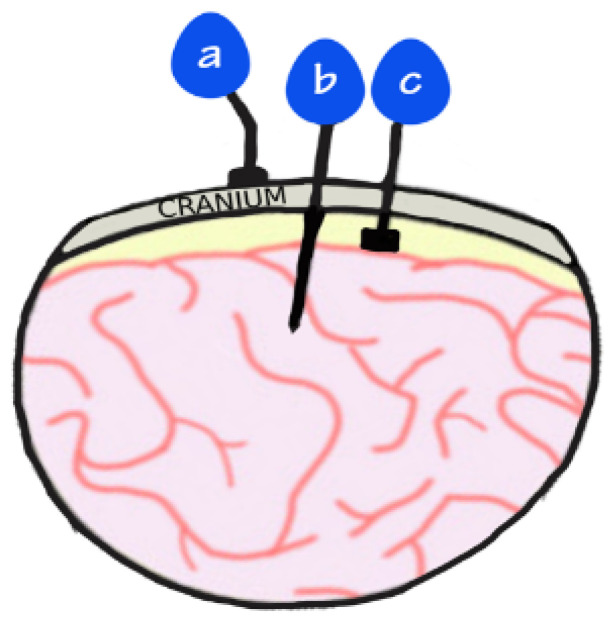
Image demonstrates placement of electrodes: (a) EEG that sits on top of the scalp; (b) intracortical electrodes that penetrate the cortex; (c) ECoG electrodes that are placed on the cortex but do not penetrate it.

Conventional augmentative and alternative communication (AAC) devices rely on residual motor functions to provide a partial solution. In some cases, people who retain partial motor function can use specially modified peripherals (e.g., mouse, joysticks, stylus, or button box) to access such AAC devices [4]. In other, more severe cases where minimal voluntary motor control is retained, more sophisticated methods are needed to detect more subtle movements like those of the head, eye gaze tracking and blinking, allowing people to communicate by spelling out messages. However, these devices require considerable effort and are often slow, failing to restore the natural fluidity of communication [5,6]. Brain–computer interfaces (BCIs) offer a more promising approach by directly translating neural activity into external device control, bypassing damaged motor pathways [7]. A BCI sensor can be placed at various depths away from the target location, ranging from sensors placed on the scalp known as surface electroencephalogram (EEG) to intracortical recordings where microelectrodes are inserted within target locations of cortical tissue (Figure 1). Other invasive sensors include electrocorticography (ECoG), which sits on the cortical surface but does not penetrate, and stereotactic electroencephalography (sEEG), which includes depth electrodes that are able to record from deep brain structures [8]. However, the greatest drawbacks to invasive methods are the risks of surgical complications and anaesthesia, as well as the risk of postoperative infection [9]. As such, there have been limited cases, and of those that have been explored, participants are usually those that have otherwise very poor outcomes, for which an investigational device exemption is required.

Currently, patients with restricted communication abilities use augmentative and alternative communication devices. These include the use of neck and head movements or eye movements to be able to allow individuals to communicate by spelling out messages. However, this requires considerable effort and is slow [5]. This is where BCIs can have a major impact, as they can directly decode cortical activity and control external devices to enable more seamless communication. This scoping review provides an overview of intracortical BCIs that have been used to provide neurological rehabilitation in patients with impaired communication. Given the scale at which the field has advanced over the last decade, this review provides an update on what is currently possible in an academic setting and highlights important technical features.

## 2. Methods

**Figure 2:**
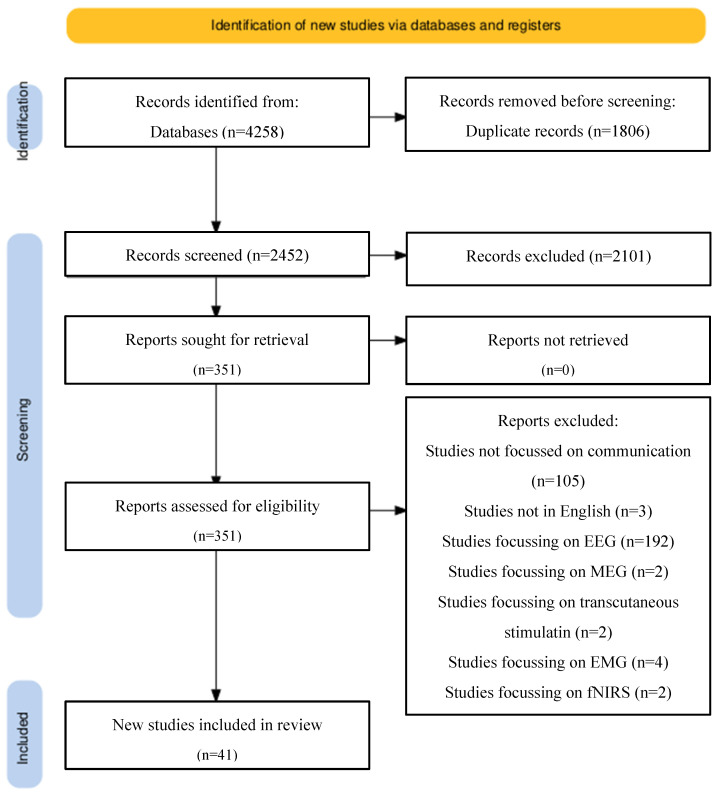
PRISMA reporting: preferred reporting items for systematic review and meta-analysis (PRISMA) flow chart explaining selection of articles in a step-wise manner as well as reasons for the exclusion of studies. Databases used included PubMed and Ovid Medline.

A comprehensive literature review was conducted based on the preferred reporting items for systematic reviews and meta-analysis guidelines (PRISMA). Ovid Medline alongside PubMed were used to search for keywords and MeSh terms including “brain-computer”, “neuroprosthesis”, “neuroprosthetics, “intracortical”, “intracortically” “brain-controlled”, “brain-machine”, “microelectrode array”, “neuroprosthesis”, “electrical neuromodulation” AND “Locked in syndrome”, “locked-in-syndrome”, “amyotrophic lateral sclerosis”, “dysarthria”, “communication”, “speech”, “Brain Hemorrhage”, “Traumatic”, “brain injury”, “head injury”, “Diffuse Axonal Brain Injury”, “trauma”, “stroke”, “cerebral infarction”, “cerebral hemorrhage”, and “cerebral vascular accident”. The full search strategy is illustrated in Figure 2.

### 2.1. Study Types

Study types including case reports or case series were included. Due to the nature of this research, there are no large-scale published reports or randomised controlled trials (Figure 2).

### 2.2. Inclusion and Exclusion Criteria

All clinical studies investigating the outcomes of invasive BCIs for communication purposes were included from January 2005 to October 2024 due to the significant progress made over the last couple of decades. This included ECoG, intracortical and sEEG electrodes that enabled patients to communicate through either a digital screen or artificial voice. Additionally, only studies that were published in English were included due to resource constraints, and only human studies were considered. Whilst animal and in vitro models have been vital for the progress that has been made in this field, we wanted to focus exclusively on human studies to assess the neurotechnologies that were closest to clinical feasibility and could avoid the at-time difficult transition from animal to human testing. As such, animal or in vitro models were excluded. Review articles, non-human studies, and studies in languages other than English were excluded. Additionally, we checked reference lists of relevant publications to identify otherwise missed studies.

### 2.3. Research Questions

This study aimed to determine the current landscape of invasive BCIs for communication. As such, we posed a few questions:

(1) What pathology did the patient cohorts present with?
(2) How successful are intracortical BCIs in restoring communication?
(3) How many electrodes were used for implantation in intracortical devices?
(4) What task did subjects have to perform for the BCI device to convert into a method of communication?
(5) How successful are ECoG devices in restoring communication?
(6) What promise do sEEG devices have in restoring communication?
(7) Which anatomical region of the brain do BCIs target to facilitate communication?

## 3. Results

### 3.1. Patient Profile

Most of the studies occurred in the United States (US) (31/41), 5 studies were published in the Netherlands and 2 from Israel. China, Canada, and the United Kingdom each published one study (Table 1). No studies were performed on children. In studies where patients had epilepsy or movement disorders, BCIs were implanted as an opportunistic research experiment. In the remaining studies, patients either had tetraplegia or locked-in syndrome.

### 3.2. Intracortical Implants

A total of 15 studies examined the effects of the implantation of intracortical BCIs into patients [10,11,13,21,25,28,30,32,34,35,36,38,40,42,45,46], and all electrodes used were supplied by BlackRock Neurotech (Table 2). Notably, none had functional movement in the upper and lower limbs. There were 29 separate cases on the patients involved. In total, 10 looked at patients suffering from ALS [11,21,34,35,36,38,45], 6 assessed patients with brainstem stroke [35,38,40,42,45,46], and 13 included patients with spinal cord injury [10,13,25,28,30,32,34,36].

Patients with spinal cord injuries had C4–C6 injuries (Table 2) that left them with poor control of their extremities. The monitoring of neural instability prompting recalibration was demonstrated, which can make monitoring patients more efficient [10]. Additionally, in two studies, the supramarginal gyrus (SMG) was also shown as an anatomical target, suggesting the SMG not only holds an internalised representation of vocalised and internal speech, but the neural signals can also be used for speech BMIs [13,25]. Wireless implantation that allowed patients with severe motor impairment to live in the community was demonstrated [30].

When controlling a cursor on the screen to select targets, it is possible to achieve up to approximately 24 characters per minute by optimising user interface. Simply adapting a keyboard that has closer targets such as the opti-II keyboard significantly increases the speed of selection [36]. This is particularly relevant as the surveying of ALS patients suggests that 72% would be satisfied with a speed of 15–19 correct characters/min [50]. The motor cortex can be targeted for the movement of a cursor through motor imagery, with users achieving a minimum syllable recognition accuracy of 54.7% and similarly a minimum word decoding accuracy of 61.5%, with variations among patients perhaps suggestive of the need to optimise other factors that can affect performance. Further research is required to elucidate these results [32].

Additionally, the SMG has been shown to be an important target for decoding words, with chronic implantation achieving up to 79% average decoding accuracy, and is found to be a common anatomical landmark for internal and vocalised speech, as well as grasp motor imagery, suggesting dual purposes [25].

Intracortical implants were also used for patients suffering from ALS (Table 3). The Revised Amyotrophic Lateral Sclerosis Functional Rating Scale (ALSFRS-R) score ranged from 6 to 23 [11,34,35,36]. The ALSFR scale demonstrates the severity of ALS on a scale of 0–40, with a higher scale demonstrating greater retained function. Two cases looked at patients attempting to speak [11,21], whilst seven [34,35,36,38,45] looked at patients attempting to move a cursor. Six cases involved the implantation of a 96-channel electrode [35,36,38,42,45], one study implanted a 4 × 64 channel electrode implant [11], and one study implanted 2 × 96 electrodes [34]. Earlier studies examined the ability of patients with ALS to communicate by controlling a cursor using an implanted BCI. One study showed patients achieving a word selection of 8.3–25.3 words per min and an ability to communicate using common digital solutions such as email, internet browsing, etc. [34]. In another study, patients achieved 6.88 correct characters/min but demonstrated no need for recalibration over 138 days [35]. Auto-calibration being comparable to a standard decoder is also demonstrated in two more patients in a separate study [38]. The importance of keyboard selection is also demonstrated as patients were able to achieve an increase in speed of up to 1.3× when using a Opti-II keyboard compared to a QWERTY keyboard [36]. However, this has proven to be slow and requires considerable effort. Later studies that attempted to decode words proved to have a much greater accuracy and speed. In one study, patients achieved an accuracy of 97.5% using a 125,000 word vocabulary [11]. Another study showed a patient being able to communicate at a rate of 62 words per minute, which is significantly higher than the results obtained with cursor control [21].

Patients with brainstem strokes also have a similar functional basis to those with ALS and spinal cord injuries. Most of these studies looked at motor imagery where patients were asked to move a cursor on a screen (Table 4). The focus of the implantation was in the precentral gyrus [35,40,42,45,46], with the aim of taking advantage of the neural recordings that occurred in the arm/hand area of the motor cortex. While high accuracy can be achieved, low speed hampers the performance. Self-calibration can be used to avoid the need for constant calibration, and results comparable to a standard decoder can be achieved [38]. One study demonstrated the potential of patients choosing approximately 3 correct characters/min to communicate [35], whilst another had rates of 2.7–8.6 correct selections/min but also demonstrated successful results 1000 days after implantation [42,45]. The importance of choosing a correct keyboard design was again demonstrated with radial keyboards outperforming the QWERTY keyboard [40].

### 3.3. ECoG-Based Studies

ECoG arrays were initially used in patients with epilepsy, and surgeons placed them according to patient needs as oppose to research purposes. However, the discoveries from these have led to significant improvements in our understanding of how speech is modulated in the brain [19,22,24,47,49]. Additionally, the studies also demonstrated that patients were able to control virtual keyboards even with ECoG devices outside of the language centre [51]. Anatomical regions including the ventral sensorimotor cortex (vSMC), superior temporal gyrus (STG) and inferior frontal gyrus (IFG) were demonstrated to be areas involved in language production when patients only mime the sounds [33]. Gesture prediction was higher than phoneme prediction overall, but anatomically, gesture prediction was substantially more accurate in the posterior areas of the cortex (corresponding largely to the primary sensorimotor and part of premotor cortices), whilst in more anterior areas, the performances for both gesture and phoneme prediction were more similar [39].

ECoG-based BCIs were also implanted in patients with ALS and stroke (Table 5). In patients with ALS, subdural electrodes were places over the sensorimotor cortex [14,16,18] and prefrontal cortex [16]. On the other hand, a pontine stroke patient had a high-density (hdECoG) array covering the left precentral gyrus, postcentral gyrus, posterior middle frontal gyrus, and posterior inferior frontal gyrus [29], while another pontine infarct patient had subdural electrodes covering the posterior aspect of the middle frontal gyrus, precentral gyrus, and anterior aspect of the postcentral gyrus, as well as the dorsal posterior aspect of the inferior frontal gyrus [23], and the final patient with a bilateral pontine stroke had implantation in the region of the dorsal posterior aspect of the inferior frontal gyrus, posterior aspect of the middle frontal gyrus, precentral gyrus, and anterior aspect of the postcentral gyrus [12]. In contrast to intracortical implants, ECoG arrays covered larger areas of the brain, although a study suggested the control of a cursor through a single subdural electrode strip with four electrodes measuring 4 mm each [41]. ECoG arrays are also able to convert attempted speech production directly into words spelled at a rate of 29.4 characters/min and a character error rate of 6.13% [23], whilst another study demonstrated the successful decoding of bilingual speech for both English and Spanish phrases [12], suggesting that the speech BCI can be used for languages other than English. The impact of recurrent neural networks to convert attempted speech into acoustic speech is demonstrated [14].

Furthermore, one study demonstrated the significance of the dorsolateral prefrontal cortex (dlPFC) as an anatomical target for BCIs for cursor control [43]. The potential benefits of using dlPFC in BCIs include providing an alternative target in cases where surgeons are unable to utilise the sensorimotor signals. This approach offers an alternative communication pathway for individuals with LIS who may have difficulty modulating sensorimotor activity due to their neurological conditions. Additionally, both participants reported finding dlPFC-based BCI control less mentally taxing than sensorimotor-based control.

Using predictive algorithms also helped with speed as spelling initially took 52 s per letter, but the time required dropped to 33 s per letter when word prediction was used. Crucially, the system could be used when the existing mode of communication failed as seen whenever she went outside, where lighting conditions made eye tracking impossible. The patient also expressed greater satisfaction with the BCI than eye-tracking system [37].

### 3.4. SEEG-Based Studies

sEEG has also been utilised as a tool in this domain. A particular advantage of this method is that sEEG devices have already been successfully implanted in patients for multiple years, as seen in the treatment of Parkinson’s disease [52].

The electrode size is approximately akin to that of surface EEG devices, although there may be a limit to the number of electrodes used to record signals for desired functional outcomes [15,17,26]. One study found a logarithmic relationship between the number of neurons that the electrodes recorded from and decoding accuracy. This means that increasing the number of neurons improved the accuracy, but the gains were greater with smaller neuron counts. The finding suggests that there is a point of diminishing returns when the number of recorded neurons for decoding is increased. The left Vim exhibited involvement in all three aspects of speech: production, perception, and imagery. While speech production decoding yielded the highest accuracies, likely because of the targeting of motor areas within the left Vim, the high accuracies for perception and imagery (96% and 80%, respectively) suggest that the left Vim plays a role beyond motor control in speech processing. The study discovered that vowels ‘e’ and ‘u’ were more frequently confused during decoding than other vowels, suggesting these vowels might share similar neuronal representations in the left Vim. This finding could inform future research on vowel encoding and decoding in the thalamus and contribute to refining decoding algorithms [15]. Additionally, a BCI speller using only three electrodes placed over the middle temporal visual area was able to achieve a speed of 12 characters/min, comparable to other BCIs controlling cursors [24]. For deep brain stimulation electrodes implanted in the subthalamic nucleus, it may also be possible to decode speech information from the electrical activity of single neurons in the subthalamic nucleus of patients with Parkinson’s disease. One study showed the accurate decoding of vowels during speech production (100% accuracy), speech perception (96% accuracy) and speech imagery (88% accuracy). Neuronal activity could therefore accurately predict vowel sounds that participants produced, perceived or imagined [26]. Other targets of speech decoding include the posterior hippocampal region [44]. In this study, the authors demonstrated the synthesised output corresponded in real time with utterance timings, suggesting reliable audible speech generation. However, reconstructed audio was not intelligible [27], seemingly because of using simple decoding methods. However, the study used simple decoding methods. Future studies that incorporate the use of deep learning-integrated decoding methods could provide clearer audio output. Nonetheless, this study is important in suggesting that sEEG targeting the hippocampal region can be utilised for speech production and can provide a platform on which future studies can build upon.

## 4. Discussion

**Figure 3:**
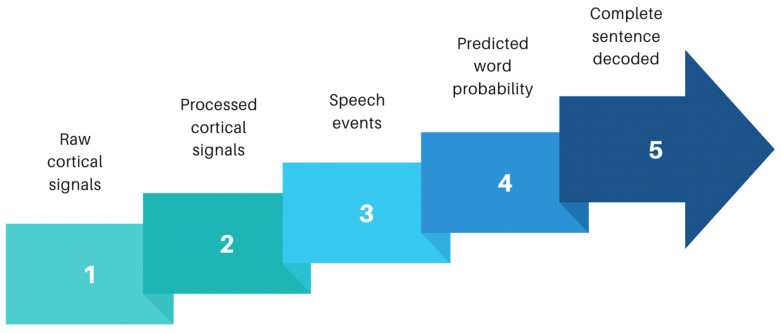
Signal processing sequences for sentence decoding: adapted from Moses and colleagues [29]. Sequential analysis from collection of raw cortical data to output of complete sentences. Raw broadband cortical signals are initially picked up by electrodes placed over the speech sensorimotor cortex. Neural signals are processed to remove noise and form meaningful processed cortical signals. Computation algorithms can then convert these processed signals into speech events by detecting speech patterns to form words. Using deep neural networks improves the success probability, and predicted words sequenced together alongside probabilistic modelling packages can allow complete sentence decoding. Sentence structures can be fed into output devices such as avatars or speech articulators to allow meaningful communication in a more natural manner.

This review focused on intracortical BCIs used for communication. Whilst earlier studies demonstrated the use of cursor control to allow the selection of characters on a screen (Figure 2), later studies have gone a step further and allowed direct speech-to-text and speech-to-audio conversion (Figure 3). However, whilst these allow users to communicate at a rate of approximately 62 words/min [21], it still falls short of natural speech production which averages 120–150 words/min. For patients, this potentially means quicker and more seamless communication, although to achieve this, the BCI package must also incorporate modern computing methods such as the use of deep neural networks and engineering solutions that allow higher-fidelity electrodes to facilitate better signal pick-up [20].

**Figure 4:**
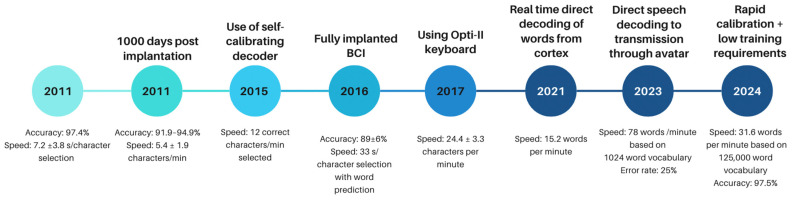
Timeline showing major advances in the field of invasive BCIs for communication. There has been a big leap forward in the rate of speech production due to direct cortex to speech production as opposed to the earlier studies that relied on cursor control. There remains heterogeneity in variables that authors choose to publish, which makes it difficult to compare outcomes.

Non-invasive technologies such as EEG, fMRI, and magnetoencephalography (MEG) do not require surgical implantation. fMRI has high spatial specificity, allowing anatomical localisation, but its low temporal resolution means that tracking neural changes at the millisecond level is difficult because of the lag in cerebral blood flow, and it also currently requires access to an MRI scanner [53]. However, MEG and EEG suffer from low spatial specificity although they have high temporal resolution [54,55]. From a practical perspective, EEG offers portability and can therefore be translated to patients in the real world. However, difficulty associated with diminishing noise means there is likely going to be a limit to scaling the accurate decoding of large vocabularies and longer speech segments. As such, invasive BCIs offer an attractive solution to overcome these deficiencies from non-invasive devices (Figure 4).

Invasive BCIs, which are separated into ECoG, intracortical, and sEEG, offer distinct advantages and disadvantages, and an ad hoc approach depending on patient needs may be beneficial. ECoG electrodes can offer high spatial resolution, typically at the millimetre scale. Furthermore, they allow the possibility of a large area to be covered, as the number of electrodes can often be tailored from dozens to hundreds of electrodes. On the other hand, sEEG typically provides sparser coverage but can record from deeper structures including limbic structures. Additionally, ECoG is often implanted to cover the unilateral recording of a cerebral hemisphere, whilst sEEG can be implanted bilaterally [56,57].

Recording from the arm/hand area of the motor cortex during controlling a cursor for communication has been well demonstrated, although the greatest disadvantage is slower speed. To improve speed, research has shifted towards the direct interpretation of speech from cortical recordings, and indeed, high-performance speech decoding is possible from recordings in the anterior precentral gyrus [11,21,32].

Anatomically, because there are multiple regions of the brain that are involved in speech production, there may exist multiple targets for implantation, although further research is required to elucidate the extent to which each region can provide speech rehabilitation. Input to the vSMC and middle precentral gyrus (midPrCG) is received from the superior temporal gyrus and supramarginal gyrus. Somatotopically arranged neural populations sit along the vSMC and middle precentral gyrus, forming the corticobulbar system [58,59]. They play an important role in controlling vocal tract articulators including the tongue, jaw, lips, and larynx, which work in a coordinated manner to produce speech driven by expired air. Dorsally located is the region that controls hand movement [60,61], although targeting this region is more appropriate for the on-screen control of cursors. Given the large number of regions that are involved in speech production, targeting the intact regions may be a viable method for speech decoding. Placing an ECoG array therefore provides an advantage given the large coverage that is possible including the vSMC, superior temporal gyrus, and midPrCG with a single array.

Studies have also demonstrated the at-home use of these device [30]. Along with decoders that can auto-calibrate [38], it allows patients to live a more normal life in an environment in which they are more comfortable. As such, translation to a real-life clinical scenario is more likely with devices that are unrestrictive in terms of location.

Additionally, it is evident that accurate decoding using modern solutions including artificial intelligence will substantially improve BCI clinical outcomes [38]. In addition, studies should focus on improving the user experience, which includes the interface and ease with which patients can interact with the device. This will likely lead to significant improvements in speed and accuracy [36].

An important consideration that should be acknowledged is the surgical risks that are also associated with the implantation of invasive BCI devices (Table 6). Whilst surgical implantation techniques will likely be optimised to reduce invasiveness and minimise intraoperative risks to patients, current methods of implantation involve a craniotomy approach to expose large areas of cortical tissue. Surgical risks include the risk of infection, bleeding, and damage to eloquent areas. This can also have an impact on the devices themselves as intracortical implants such as microelectrodes can elicit inflammatory responses, leading to scarring and the loss of neurons [62,63].

The long-term efficacy of BCIs is a topic of research. Inflammation after implantation likely will contribute to the chronic stability of recordings, but this could also be dependent on the implantation technique such as the disruption of microvasculature as the device is implanted. With ECoG devices, impedance will likely stabilise after several months [64,65,66]. Intracortical devices also have additional unique challenges. Surgical implantation is of greater significance as factors such as the size, material, and shape of electrodes, insertion speed, and roughness of the implantable device can lead to an acute inflammatory response [67,68,69]. Thus, immediately after insertion, neurons directly in contact can be killed, which can significantly reduce the neuron population. Subsequent glial response can further propagate the inflammatory response. Additionally, as the device is often tethered, electrodes are fixed whilst the brain can move independently, causing further damage to the brain through these micromotions. Over time, a glial scar with a size of ~100 μm can form, which creates a further barrier between the electrode and the brain, increasing the space between neurons and electrodes [70,71,72]. This can lead to worsening signal quality, through neural displacement, electrical insulation, and the modulation of neighbouring neurons. During this phase, there is typically an increase in electrical impedance, although this stabilises over the following months. However, despite the decline in signal quality, the impact on users’ functional performance may not be linear, as participants are able to maintain high BCI performance many months/years after insertion [66].

However, patient pathology may also contribute to worsening signal quality. This is particularly evident in cases where the brain will atrophy such as in ALS. If the electrodes are fixed to the skull, the sensors can migrate from the region of interest if the brain atrophies as with ALS, thereby limiting the functional use of the BCI [16]. Therefore, the clinical translation of devices will benefit from wireless implantation devices.

Lastly, the studies lack homogeneity when discussing different tasks performed by a user which makes comparisons difficult. The choice of behavioural tasks on brain performance is an important consideration in neurotechnology research because the choice of task can have a large impact on decoding accuracy and speed and overall system reliability, which greatly affects patient performance. More complex tasks will also require greater cognitive effort. Additionally, the nature of the task is also important. Using motor imagery whereby users mentally rehearse movement to invoke event-related desynchronization (ERD) and synchronisation (ERS) patterns in the sensorimotor cortex involves different levels of training and mental effort compared to speech decoding, but a comparison between these tasks is difficult without standardised behavioural task protocols. This includes using metrics such as session duration, the number of trials, rest intervals, feedback mechanisms, and control conditions to account for spontaneous neural activity fluctuations. Performance metrics should also be standardised, which not only includes objective data such as characters/min and speed but also user fatigue and cognitive load. Therefore, the development of consensus guidelines for the reporting of BCI performance is required and would greatly enhance comparability and reproducibility across studies.

## 5. Ethics

As the field of BCIs evolves, a range of ethical issues has to be addressed (Table 6). Whilst this is not the focus of this review, we include it here as a point of discussion to raise awareness. Implantable BCIs pose further concerns in comparison to non-invasive technologies as the surgical risks must also be considered. Invasive BCIs have the potential to enhance the ability of users [73]. Whilst the studies performed thus far have been on patients suffering from a neurological deficit, it is possible to adapt the studies for healthy participants to facilitate connection with external devices. This can lead to, for instance, the ability to control a robotic arm or computer screen through thought. Justifying surgical risks for such procedures can be considered adjacent to cosmetic procedures that may not be life-saving but performed if patients desire it. However, the ability to record and stimulate the nervous system has wider implications including societal risks.

Enhancing individuals beyond their natural limitations can be seen by some as going against limitations set naturally. If such technology was only accessibly to select few due to limited access or high costs, it can create a divide in society where individuals who have neuroenhancement may be seen as superior or be able to have a wider repertoire of functions available to them [8]. This ties in with the concept of distributive justice. From a utilitarian perspective, if only select individuals have access to technologies that allow them the ability to enhance themselves, the large divide in society can be more detrimental than the potential positive that it can bring. This can be seen, for example, with enhancement drugs such as stimulants that allow students to gain an advantage over their peers [74].

Furthermore, as BCIs have the ability to collect neuro-data, it can provide a rich source of data for commercial uses, such as consumer targeting. Additionally, neural signals are closely aligned with an individual’s identity, and as such, the use of these data can infringe upon patient confidentiality [8,75].

Finally, as BCIs can be connected with other digital devices, it opens up the possibility of hacking and infringing upon user autonomy. This is already a concern with other implantable devices such as insulin pumps and pacemakers [76,77]. Because the brain is such a complex organ, hacking and accessing neuro-data as well as stimulating nervous tissue can have severe and unpredictable consequences.

It is therefore imperative that regulation of BCIs is given priority and continuously updated as our understanding of neuroscience improves and neurotechnology evolves.

## 6. Conclusions

BCIs provide a significant therapeutic option for patients who otherwise have limited rehabilitation recovery. However, over the last decade, the pace of this field has been rapid and has shifted from cursor control to the decoding of speech directly from the brain in a bid to improve the speed of communication. The ability to facilitate communication in a neurologically impaired patient can significantly improve their quality of life. However, significant resources are required to achieve greater results. Interpreting high-quality signals and decoding them to speech requires innovative solutions such as the incorporation of AI to reduce noise and improve language interpretation, as well as optimising the user interface to allow patients to interact better with the software in a more natural manner. However, costly innovations may run the risk of secluding patients who cannot access this technology, which can create a societal divide. As such, understanding stakeholder perspectives including patients must be central when translating this technology from the lab to the clinic. Additionally, future studies should also provide greater feedback from the users of BCI devices. In particular, the ease of use and the mental effort that is required is often missed in many studies. This will provide great insight into the rehabilitation process that is required during the post-implantation period and can provide a useful learning area for other researchers to benefit from. Having user interfaces or integrated AI modalities that make it easier for users to engage with the BCI can be as useful as improving the technical designs of the BCIs in improving patient outcomes.

User feedback is rarely reported in detail in these studies. Whilst some studies allude to improved user experience—for example, with an easier-to-use keyboard, feedback mechanism, or improved calibration strategies—a qualitative study that assesses the user experience is necessary to explore this in more depth, which includes the complete rehabilitation phase from the post-operative phase to chronic implantation. This will allow a greater understanding of the patient experience and can be used in conjunction with objective markers such as characters per minute, the speed of character selection, and recovery times.

To effectively compare studies, there needs to be homogeneity in protocol design and reporting outcomes in patients.

## 7. Limitations

There are several limitations to this study. A scoping review cannot guarantee that all studies assessing intracortical BCIs for communication are exhausted, found and analysed. The literature output is confined by the search terms that we entered, and although we attempted to keep the search terms as broad as possible, it is possible that relevant studies were missed out. This includes studies that have labelled their devices as anything other than a ‘brain–computer interface’. The focus of this scoping review was on patients suffering from the disease as opposed to healthy participants; indeed, to our knowledge there are no invasive systems being tested on non-impaired individuals. Due to the small sample size, statistical analysis was difficult to conduct. It is also important to acknowledge that there are likely multiple user factors that affect the accuracy of signal translation, making it difficult to draw meaningful conclusions that can be applied to all patients with these conditions.

## Figures and Tables

**Table 1:** Studies involving intracortical implantation for communication disorders.

Title	Year Published	Country
Measuring instability in chronic human intracortical neural recordings towards stable, long-term brain-computer interfaces [10]	2024	USA
An Accurate and Rapidly Calibrating Speech Neuroprosthesis [11]	2024	USA
A bilingual speech neuroprosthesis driven by cortical articulatory representations shared between languages [12]	2024	USA
Representation of internal speech by single neurons in human supramarginal gyrus [13]	2024	USA
Online speech synthesis using a chronically implanted brain-computer interface in an individual with ALS [14]	2024	USA
Machine learning decoding of single neurons in the thalamus for speech brain-machine interfaces [15]	2024	Israel
Longevity of a Brain-Computer Interface for Amyotrophic Lateral Sclerosis [16]	2024	The Netherlands
Speech decoding from stereo-electroencephalography (sEEG) signals using advanced deep learning methods [17]	2024	UK
Stable Decoding from a Speech BCI Enables Control for an Individual with ALS without Recalibration for 3 Months [18]	2023	USA
Distributed feedforward and feedback cortical processing supports human speech production [19]	2023	USA
A high-performance neuroprosthesis for speech decoding and avatar control [20]	2023	USA
A high-performance speech neuroprosthesis [21]	2023	USA
Direct speech reconstruction from sensorimotor brain activity with optimized deep learning models [22]	2023	The Netherlands
Generalizable spelling using a speech neuroprosthesis in an individual with severe limb and vocal paralysis [23]	2022	USA
Intracranial brain-computer interface spelling using localized visual motion response [24]	2022	China
Decoding grasp and speech signals from the cortical grasp circuit in a tetraplegic human [25]	2022	USA
Machine learning algorithm for decoding multiple subthalamic spike trains for speech brain-machine interfaces [26]	2021	Israel
Real-time synthesis of imagined speech processes from minimally invasive recordings of neural activity [27]	2021	The Netherlands
Generalizable cursor click decoding using grasp-related neural transients [28]	2021	USA
Neuroprosthesis for Decoding Speech in a Paralyzed Person with Anarthria [29]	2021	USA
Home Use of a Percutaneous Wireless Intracortical Brain-Computer Interface by Individuals With Tetraplegia [30]	2021	USA
Dorsolateral prefrontal cortex-based control with an implanted brain-computer interface [31]	2020	The Netherlands
Neural ensemble dynamics in dorsal motor cortex during speech in people with paralysis [32]	2019	USA
Speech synthesis from neural decoding of spoken sentences [33]	2019	USA
Cortical control of a tablet computer by people with paralysis [34]	2018	USA
Stable long-term BCI-enabled communication in ALS and locked-in syndrome using LFP signals [35]	2018	USA
High performance communication by people with paralysis using an intracortical brain-computer interface [36]	2017	USA
Fully Implanted Brain-Computer Interface in a Locked-In Patient with ALS [37]	2016	The Netherlands
Virtual typing by people with tetraplegia using a self-calibrating intracortical brain-computer interface [38]	2015	USA
Decoding of articulatory gestures during word production using speech motor and premotor cortical activity [39]	2015	USA
Neural Point-and-Click Communication by a Person With Incomplete Locked-In Syndrome [40]	2015	USA
Real-time two-dimensional asynchronous control of a computer cursor with a single subdural electrode [41]	2012	Canada
Neural control of cursor trajectory and click by a human with tetraplegia 1000 days after implant of an intracortical microelectrode array [42]	2011	USA
Using the electrocorticographic speech network to control a brain-computer interface in humans [43]	2011	USA
Control of a brain-computer interface using stereotactic depth electrodes in and adjacent to the hippocampus [44]	2011	USA
Point-and-click cursor control with an intracortical neural interface system by humans with tetraplegia [45]	2011	USA
Control of a visual keyboard using an electrocorticographic brain-computer interface [44]	2011	USA
A wireless brain-machine interface for real-time speech synthesis [46]	2009	USA
Robust, long-term control of an electrocorticographic brain-computer interface with fixed parameters [47]	2009	USA
Electrocorticographically controlled brain-computer interfaces using motor and sensory imagery in patients with temporary subdural electrode implants. Report of four cases [48]	2007	USA
Electrocorticography-based brain computer interface—the Seattle experience [49]	2006	USA

**Table 2:** Details on intracortical implants for spinal cord injury patients.

Age and Gender	Injury	Number of Electrodes and Anatomical Placement	Electrode Details	Behaviour Task	Outcome
37M [10]	C4 AIS-B spinal cord injury (SCI)	1 × 96 channel in hand/arm knob of dominant (left) precentral gyrus	Not reported	Attempt hand/finger movement to move cursor	The novel decoder recalibration method quantifies and monitors instability in neural recording, suggesting when recalibration should take place.
65M [10]	C4 AIS-C SCI				
33M [13]	C5 spinal cord injury	1 × 96 in left SMG 1 × 96 in left ventral premotor cortex 2 × 48 in left hand/arm of S1	Platinum-tipped or sputtered iridium oxide film (SIROF)-tipped	Internally and externally vocalise words	The offline accuracy of recording from the SMG was 24% and 55% for each patient. This increased with an online internal speech task as users achieved 25% and 79% decoding accuracy. This suggests significant neural representation in internal and vocalised speech at the SMG. Additionally, activity in the somatosensory cortex (S1) was present during vocalised but not internalised speech, suggesting that no articulatory movement of the vocal tract occurred during internal speech.
39M [13]	C6 spinal cord injury	1 × 64 in left SMG, 1 × 64 in left ventral premotor cortex, 1 × 64 in primary motor cortex, 2 × 64 in left S1			
Age not reported [25]	C5 cervical spinal cord injury	1 × 96 in SMG, 1 × 96 in PMv, 2 × 48 in S1	Iridium oxide	Motor imagery of grasps meaning he imagined making the hand shapes without actually moving	The patient, who was unable to physically move his hands due to tetraplegia, was asked to perform motor imagery of the grasps. The results showed that individual grasps could be decoded from the neural activity in all three brain regions, indicating their potential as target sites for grasp BMIs. They found that the SMG could also decode spoken grasp names, suggesting its potential role in speech BMIs. This contrasted with PMv and S1, which did not have a significant classification of speech for colours or grasp names.
Age not reported [28]	C5 motor/C6 sensory ASIA B spinal cord injury	2 × 88 in hand and arm areas of motor cortex	Not reported	Motor imagery using arm to move cursor and grasp to click	This study investigated the use of transient neural responses at the onset and offset of an attempted hand grasp to provide more generalizable click control for intracortical brain–computer interfaces. The researchers developed a novel, transient-based click decoder and compared its performance to the standard sustained click decoder. This research provides evidence that a transient-based approach to click decoding can significantly improve iBCI control by enabling both discrete and sustained click functionality.
Age not reported [28]	C6 ASIA B spinal cord injury	2 × 96 implanted in hand area of motor cortex	Not reported		
63M [30]	C4 AIS-C spinal cord injury	2 × 96 implanted in left (dominant) precentral gyrus	Platinum tips	Point and click of commercial apps using cursor at home	The study found that the wireless system could effectively record and decode neural signals, allowing participants to control a computer cursor and a tablet computer. The performance of the wireless system was comparable to that of the wired system in terms of accuracy and speed. The successful implementation of the wireless system in a home setting marks a significant step towards developing more practical and user-friendly assistive technologies for people with severe motor impairments.
35M [30]	C4 AIS-A SCI		Platinum tips		
64M [32]	C4 AIS-C SCI	2 × 96 implanted in dorsal hand knob area of left (dominant) motor cortex	1.5 mm electrode	Patient attempted speech and orofacial movement when prompted	The study shows the hand knob motor area of the motor cortex to also be responsible for speech production. Patient 1 had an accuracy of syllables of 84.6% and word decoding of 83.5%. Patient 2 had an accuracy of syllables of 54.7% and 61.5% of word decoding.
56M [32]	C4 AIS-A SCI		1.5 mm electrode		
63M [34]	C4 ASIA-C	1 × 96 implanted in hand and arm area of dominant (left) motor cortex	1 mm electrode	Motor imagery using hand/arm flexion to control cursor	The study looked at three patients including two ALS and one spinal cord injury patient using a point and click device on a commercial tablet. Patients were able to select 8.3–25.3 words per minute but also used other mediums such as email, chat, browsing the web, and accessing weather, news, music, and videos.
63M [36]	C4 ASIA-C	2 × 96 implanted into upper extremity area of dominant (left) motor cortex	1.5 mm intracortical silicon microelectrode	Move cursor on screen to type	When free typing, one patient was able to achieve 24.4 ± 3.3 characters per minute. Two patients using Opti-II keyboard performed quicker than when using a qwerty keyboard. One patient performed quicker using an abcdef keyborard than when using opti-II, but the patient had minimal typing experience beforehand.

**Table 3:** Details on intracortical implants for ALS patients.

Age and Gender	Extent of ALS	Number of Electrodes Anatomical Placement	Electrode Details	Behaviour Task	Outcome
45M [11]	Tetraparesis + severe dyarthria ALS functional rating scale revised (ALSFRS-R) score is 23	256 electrodes in left precentral gyrus—patient was confirmed to be left hemisphere language-dominant by fMRI	4 microelectrodes with each measuring 3.2 × 3.2 mm in size and electrode depth 1.5 mm	Attempt to speak	The BCI achieved an accuracy of 99.6% using a 50-word vocabulary. With training, the accuracy was 97.5% using a 125,000-word vocabulary. Arrays in the ventral premotor cortex and middle precentral gyrus contributed most to decoding accuracy.
51F [34]	ALSFRS-R score is 14—retained speech and dexterous movement of wrist and some fingers	96-channel electrodes in hand area of dominant (left) motor cortex	1 mm electrode length, 4 × 4 mm	Motor imagery using hand/arm flexion to control cursor	The study looked at three patients using a point and click device on a commercial tablet. Patients were able to select 8.3–25.3 words per minute but also used other mediums such as email, chat, browsing the web, and accessing weather, news, music, and videos.
51M [34]	ALSFRS-R score is 6—retained speech but minimal movement in hands/fingers	2 × 96 electrodes in hand area of dominant (left) motor cortex	1.5 mm electrode length	Motor imagery using hand/arm flexion to control cursor	
Age not reported [35]	ALSFRS-R score is 16—had tracheostomy and on-demand ventilation. She could speak but had limited hand mobility	96 channel electrode into arm area of dominant precentral gyrus	Not reported	Move cursor on screen	The study looked at intracortical local field potentials where the patient averaged one word per minute without the need for recalibration and sustained performance over several months. The study lasted for 138 days. Spelling rates of 6.88 correct characters/minute allowed the patient to also type messages and write emails.
51F [36]	ALSFRS-R score is 16—retained dexterous movement of hand and wrist	96 channel electrode into hand area of dominant (left) motor cortex	1.0 mm silicon electrode	Move cursor on screen to type	When free typing, the patient was able to achieve 24.4 ± 3.3 characters per minute using an Opti-II keyboard. This was 1.3 times quicker than when using a QWERTY keyboard.
54M [36]	ALSFRS-R score is 17—very limited movement in fingers	96 channel electrode implanted into hand area of dominant (left) motor cortex	1.5 mm silicon electrode	Move cursor on screen to type	The patient performed quicker using an ABCDEF keyboard than when using a QWERTY keyboard. However, the patient also had limited typing experience beforehand.
51F [38]	Not reported	96 channel electrode implanted into hand/arm knob area of dominant motor cortex	1.0 mm electrodes	Selecting character on screen	The auto-calibration of the decoder using a retrospective decoder produced comparable accuracy (12 characters correct per minute) as a standard decoder (11.4 correct characters/minute). Even with longer trials involving self-typing sessions, i.e., 1–2+ h, typing rates remained high.
58M [38]	Not reported		1.5 mm electrode		
37M [45]	Paralysis	96 channel electrode implanted into arm area of dominant motor cortex	Not reported	Move cursor by imaging being able to control the cursor and click by imaging right hand opening	In total, 52.6% of targets were selected correctly but failed only due to time-out. The false-click average was one per trial.
67F [21]	Patient has bulbar onset ALS. Whilst she retains some limited orofacial movement and can vocalise, she has an inability to produce intelligible speech	2 × 64 channel electrode implanted ventral premotor cortex (area 6v) and 2 × 64 channel electrodes in area 44	3.2 mm arrays	Attempt at speech	The patient was able to communicate at a rate of 62 words per minute, which was significantly higher than previous approaches. She had a 23.8% error rate on a vocabulary consisting of 125,000 words.

**Table 4:** Details on intracortical implants for brainstem stroke patients.

Age and Gender	Stroke Pathology	Number of Electrodes Anatomical Placement	Electrode Details	Behaviour Task	Outcome
Age not reported [35]	Brainstem stroke leading to locked-in syndrome	96-channel electrode into arm area of dominant precentral gyrus	Not reported	Moving cursor on screen	The study looked at intracortical local field potentials where the patient averaged one word per minute without the need for recalibration, and sustained performance over several months. Study lasted for 76 days. Spelling rates of 3.07 correct characters/minute allowed patient to also type messages and write emails.
57F [38]	Not reported		1.5 mm electrode tips	Selecting character on screen	The auto-calibration of the decoder using a retrospective decoder produced comparable accuracy (12 characters correct per minute) as a standard decoder (11.4 correct characters/minute).
66M [38]	Not reported		1.5 mm electrode tips		
58F [40]	Bilateral pontine infarction	96-channel implanted in the arm/hand area of her motor cortex	Not reported	Moving cursor on screen by attempting to move dominant hand at wrist	Using a radial keyboard led to an improvement of 65% in correct characters selected per minute and outperformed the QWERTY keyboard in all tasks. The patient was also able to use the keyboard to enter google chat and communicate at a rate of 8.1 correct characters per minute
56F [42]	Brainstem stroke leading to anarthria and tetraplegia	96-channel implanted in arm area of motor cortex	Not reported	Moving cursor on screen by attempting to move dominant right hand and right hand grasp to select	After 1000 days post implantation, the BCI was still working well. The patient was able to successfully select 2.7–8.6 correct selections/minute on a screen at a success rate of 91.9–94.9% over 5 days.
26M [46]	Brainstem stroke leading to locked-in syndrome	Neurotrophic electrode implanted into precentral gyrus	Single 3-wire electrode	Attempted speech patterns were wirelessly transmitted and converted in real time using a Kalman filter-decoder	The participant significantly improved their performance of a vowel production task over a 1.5 h period involving 34 or fewer vowels, increasing the average accuracy from 45% to 70%.
55F [45]	Brainstem stroke leading to tetraplegia	96-channel electrode implanted into arm area of dominant motor cortex	Not reported	Moving cursor by imaging being able to control the cursor and click by imaging right hand closing	In total, 97.4% of the targets were selected correctly but failed only due to time-out. The false-click average was 0.74 per trial. It took the patient 7.2 ± 3.8 s seconds to move the cursor over a distance of 12 cm.

**Table 5:** ECoG implantation in patients to aid communication.

Age and Gender	Pathology	Number of Electrodes Anatomical Placement	Behaviour Task	Outcome
60M [14]	ALS: ALSFRS-R score of 26: Patient had primarily disability of bulbar and upper extremity muscle, resulting in mostly unintelligible speech	Two 8 × 8 subdural electrodes covering ventral sensorimotor cortex and representative dorsal laryngeal area using platinum–iridium disc electrodes covering 36.6 mm × 33.1 mm	Patient was tasked with reading aloud words in a closed vocabulary of 6 words	Using a recurrent neural network, the BCI was able to produce acoustic speech that included the characteristics and natural pacing of the patient’s speech. Native English speakers could interpret the attempted words with 80% accuracy from the synthesised speech.
35F [48]	Epilepsy with right anterior temporal lobe lesion	Subdural in right temporal lobe	Motor imagery to move cursor on screen	Cursor control can be achieved with minimal training. In this study, participants trained for 45 min in a day for a total of 2–7 days.
43M [48]	Epilepsy with left temporal lobe tumour	Subdural in left temporal lobe		
18F [48]	Epilepsy with left temporal lobe mass	Left perisylvian region		
60F [48]	Medically intractable facial pain	Right primary motor cortex		
58F [16]	ALS: ALSFRS-R score of 1	2 electrode strips over the dorsolateral prefrontal cortex and 2 over the sensorimotor cortex	Control of a cursor on a monitor and clicking	This was an update on a previous study where the patient had used the BCI to communicate with family and caregivers. This included calling for medication or requesting airway suction. After approximately 6 years of use, the signals’ quality started to decrease, coinciding with atrophy in frontal and parietal brain volume, leading to increased distance between strips (that were attached to the skull) and cortex.
61M [18]	ALS patient with bulbar dysfunction, leading to severe progressive dysarthria and dyspnoea	2 × 64 channel subdural with each strip covering a surface area of 36.66 mm × 33.1 mm over the ventral sensorimotor cortex	Patient read single text commands aloud or mimed them as they appeared on a computer monitor	Speech was accurately decoded with a median accuracy of 90.59% over a 3 month period without the need for recalibration. No adverse effects were also observed from implantation throughout the 3-month period.
36M [12]	Bilateral pontine stroke: patient was left with severe spastic quadriparesis and anarthria	hdECoG array implanted subdurally using a total of 128 electrodes and was centred to sample from dorsal posterior aspect of inferior frontal gyrus, posterior aspect of middle frontal gyrus, precentral gyrus, and anterior aspect of postcentral gyrus	Patient attempted to speak words throughout the tasks	The decoding of bilingual speech led to a median word error rate of 25.0% across online testing blocks for both English and Spanish phrases, demonstrating the system’s ability to decode intended speech in both languages. The system could freely decode the intended language with a median accuracy of 87.5% based on neural features and the differential linguistic context built throughout a phrase. A comparison between the speed of this BCI and the participant’s previous communication method, an augmentative and alternative communication (AAC) interface that used residual head movements to spell words, was also made. The BCI achieved a median speed of 21 words per minute, which was considerably faster than the participant’s AAC rate of 3 words per minute
47F [20]	Pontine infarction with left vertebral artery dissection and basilar artery occlusion—patient is unable to articulate intelligible words	hdECoG array with 253 electrodes implanted to cover regions associated with speech and language including left middle aspect of the superior and middle temporal gyri, the precentral gyrus and the postcentral gyrus	Activation of neurons was recorded with attempts to silently voice words from 249 randomly selected words by moving orofacial muscles including that of the tongue, lips, and jaw.	The study involved multiple stages of recording signals for word production. The system decodes the neural signals and displays the intended words as text on a screen. Speech Audio: The system synthesises audible speech from the participant’s brain activity. Facial Avatar Animation: The system animates a virtual avatar to accompany the synthesised speech, creating a more embodied communication experience. The avatar can display both speech-related and non-speech facial gestures, including emotional expressions.
36M [23]	Extensive pontine infarct leading to severe spastic quadriparesis and anarthria	hdECoG with 128 electrodes covering the left hemisphere associated with speech production. This includes the posterior aspect of the middle frontal gyrus, the precentral gyrus, and the anterior aspect of the postcentral gyrus, as well as the dorsal posterior aspect of the inferior frontal gyrus.	Patient attempted to start speaking	The primary goal was to assess if the participant could use silent attempts to speak to control the BCI and spell out intended messages from a 1152-word vocabulary. The system achieved a median character error rate of 6.13% and a median word error rate of 10.53% during the copy-typing task. The median spelling rate was 29.4 characters per minute and 6.9 words per minute, exceeding the participant’s typing speed with his existing assistive device. Silent Control: This study is the first to demonstrate successful sentence decoding from silent speech attempts, paving the way for communication restoration in individuals with complete vocal tract paralysis.
36M [29]	Pontine stroke associated with dissection of right vertebral artery	128 electrodes placed in subdural space over left sensorimotor cortex including left precentral gyrus, postcentral gyrus, posterior middle frontal gyrus, and posterior inferior frontal gyrus	Attempted speech production	The BCI system successfully decoded full sentences from the participant’s cortical activity in real time. The median rate of decoding was 15.2 words per minute, with a median word error rate of 25.6%.
58F [43]	ALS patient with ALSFRS-R score of 2	Subdural placement in left prefrontal cortex and left sensorimotor cortex	Cursor movement	The study demonstrated the use of the dorsolateral prefrontal cortex (dlPFC) as an anatomical target to control a cursor in a one-dimensional BCI task. This task involved moving a cursor up or down by either performing serial subtraction or resting.
39F [43]	Pontine stroke leading to tetraplegia			
58F [37]	ALS patient with ALSFRS-R score of 2	Subdural electrodes placed over the hand region of the left motor cortex, and left prefrontal region	Cursor movement	The study looked at communication for an ALS patient with LIS. The patient used the BCI for 262 days. The patient was able to achieve an accuracy of 89 ± 6% of the time, with the subjective mental effort required decreasing from an initial 5 to 2.8 out of 5.
36F, 49M, 45F, 48F [31]	Epilepsy	Subdural positioned strip consisting of 64 (8 × 8) electrodes positioned on left lateral surface	Patients controlled a cursor by expressing a series of phonemes	Regions coding for phonemes included Wernicke’s area (BA 40), the auditory cortex (BA 42 and BA 22), premotor cortex (BA 6), and sensorimotor cortex (BA 3). All patients achieved accuracy greater than 69% after 4 to 15 min of closed-loop control experiments when deciphering phonemes.

**Table 6:** Left hand column lists major surgical considerations for invasive BCIs. Right hand column lists the major ethical categories for invasive BCIs.

Surgical Considerations	Ethical Considerations
Infection Bleeding Iatrogenic damage Inflammation Anaesthetic complications Postoperative pain Lengthy rehabilitation	Legal justice: Regulatory agencies control the introduction of neurotechnologies into trials and clinical practice. Once on the market, continuous safety monitoring as well as ethical issues arising on cases of malpractice, hacking, and the mis-use of data will remain a subject of concern. Distributive justice: Neurotechnologies that are used for enhancement purposes may create a societal divide where select individuals are able to achieve improved characteristics such as strength, cognition, or connection to external devices. Autonomy: The stimulation of cortical tissue can lead to changes in thought patterns, and as such, there is plausibility for altered decision making under the influence of neurotechnologies.
